# High-altitude glacier archives lost due to climate change-related melting

**DOI:** 10.1038/s41561-023-01366-1

**Published:** 2024-01-26

**Authors:** C. J. Huber, A. Eichler, E. Mattea, S. Brütsch, T. M. Jenk, J. Gabrieli, C. Barbante, M. Schwikowski

**Affiliations:** 1 Laboratory of Environmental Chemistry, Paul Scherrer Institut, Villigen, Switzerland; 2 Department of Chemistry, Biochemistry, and Pharmaceutical Sciences, University of Bern, Bern, Switzerland; 3 Oeschger Centre for Climate Change Research, University of Bern, Bern, Switzerland; 4 Department of Geosciences, University of Fribourg, Fribourg, Switzerland; 5 Institute for Polar Sciences, National Research Council, Venice, Italy; 6 Department of Environmental Sciences, Informatics and Statistics, Ca’ Foscari University of Venice, Venice, Italy

**Keywords:** Climate-change impacts, Cryospheric science

## Abstract

Global warming has caused widespread surface lowering of mountain glaciers. By comparing two firn cores collected in 2018 and 2020 from Corbassière glacier in Switzerland, we demonstrate how vulnerable these precious archives of past environmental conditions have become. Within two years, the soluble impurity records were destroyed by melting. The glacier is now irrevocably lost as an archive for reconstructing major atmospheric aerosol components.

## Abstract (web summary)

Information on past environmental conditions stored within high-altitude glaciers is being lost due to accelerated melting associated with climate change, according to ice core analysis from a Swiss glacier.

## Main

High-altitude glaciers are unique natural archives, enabling us to reconstruct past changes in climate and environment. They contain regional information about atmospheric composition, temperature^1,2^, precipitation^3^, drought events (mineral dust)^4,5^, forest fires^6^, industrial pollutants^7^ and vegetation (pollen)^8^. Of particular interest are the pre-industrial to industrial aerosol records, which allow the placement of recent human-induced changes into a longer-term perspective. Aerosol particles have a short atmospheric lifetime of about one week and show highest concentrations closest to the sources. High-altitude glacier ice cores facilitate the reconstruction of the regional aerosol signal, which is required to constrain model simulations of global anthropogenic aerosol radiative forcing^9^. However, these archives of the past are endangered by recent global warming, which is causing substantial mass loss of mountain glaciers^10–12^. In all regions of the European Alps, widespread glacier surface lowering is being witnessed, even up to the highest altitudes^9^. This is also affecting the accumulation zones of glaciers, where ice cores for palaeo-studies are collected. When there is net ablation at ice-core drill sites, the age of the surface, which is an important anchor point for dating, is no longer known. Additionally, the overlap with instrumental observations becomes shorter or non-existent^13,14^. Such an overlap is a fundamental prerequisite for calibrating subsequent ice-core reconstructions. The threat of glacial-archived information being lost forever is a major challenge faced by the scientific community, as it forms one of the best records of past climatic and environmental changes.

**Fig. 1:**
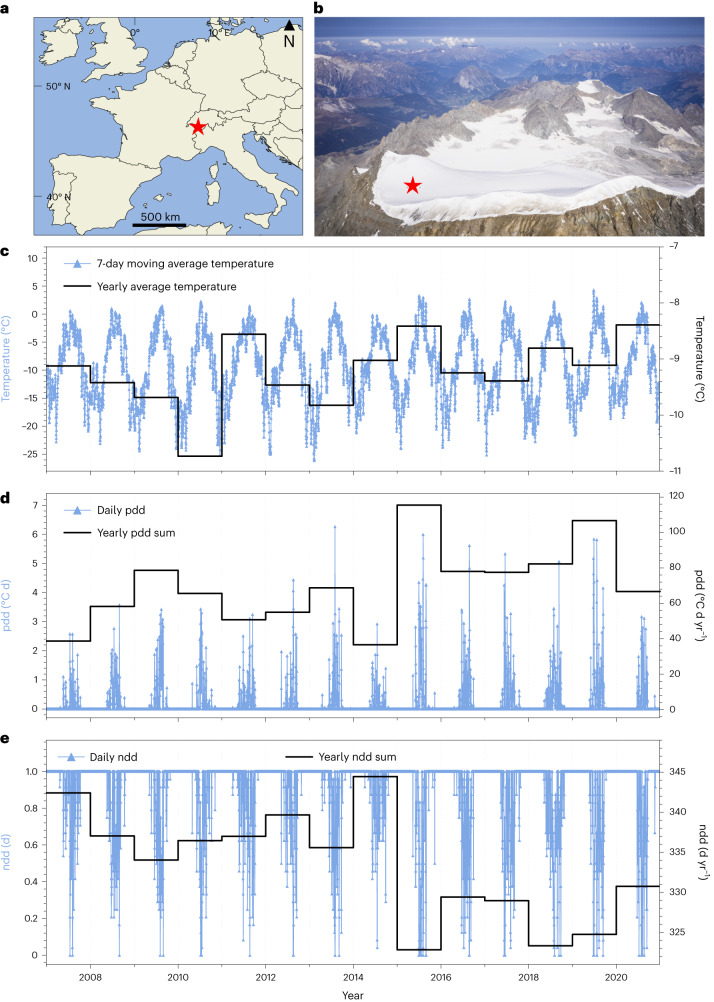
Location of the drill site and temperature data. **a**,**b**, The Corbassière glacier drill site, shown on a map of western Europe^26^ (**a**) and a picture of the Grand Combin massif (courtesy of Riccardo Selvatico; **b**). **c**–**e**, Air temperature (**c**), positive degree-day sum (pdd; **d**) and the fraction of the day with negative temperature (ndd; **e**), presented for the period 2007–2021, comparing daily and yearly averages. A different scaling was used for daily/yearly average temperatures in **c**.

We illustrate that, in the Alps, even at altitudes around 4,100 m above sea level (a.s.l.), glaciers are on the verge of becoming unsuitable as natural palaeo-archives. We use the Corbassière glacier on Grand Combin (Fig. 1a,b) in the western Swiss Alps as an example. In the frame of the ICE MEMORY initiative^15^, the summit plateau of the Corbassière glacier was chosen as a new location complementing the existing ice-core sites in the European Alps (Col du Dôme on Mont Blanc and Colle Gnifetti on Monte Rosa). On this glacier, both winter and summer snow is preserved, leading to a higher snow accumulation rate of 0.86–0.98 m water equivalent per year (w.e. yr^−1^) than at Colle Gnifetti (0.45 m w.e. yr^−1^)^16^, where most of the winter snow is eroded by wind. At the same time, this is a lower annual snow accumulation than at Col du Dôme (0.5–2.4 m w.e. yr^−1^)^17,18^, so it was expected that this archive reaches further back in time.

**Fig. 2:**
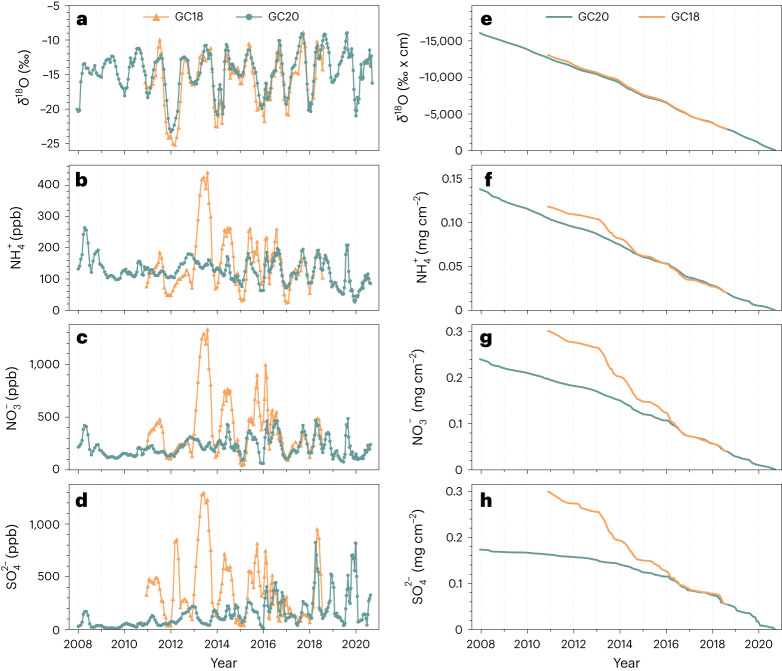
Comparison of δ^18^O and major ion concentrations from the Grand Combin 2018 (GC18) and 2020 (GC20) firn cores. **a**–**h**, δ^18^O (**a**) and concentrations of NH_4_^+^ (**b**), NO_3_^−^ (**c**) and SO_4_^2−^ (**d**) are shown, together with cumulative δ^18^O signals (**e**) and cumulative deposition fluxes of NH_4_^+^ (**f**), NO_3_^−^ (**g**) and SO_4_^2−^ (**h**), covering the periods 2011–2018 (GC18) and 2008–2020 (GC20).

**Extended Data Fig. 1:**
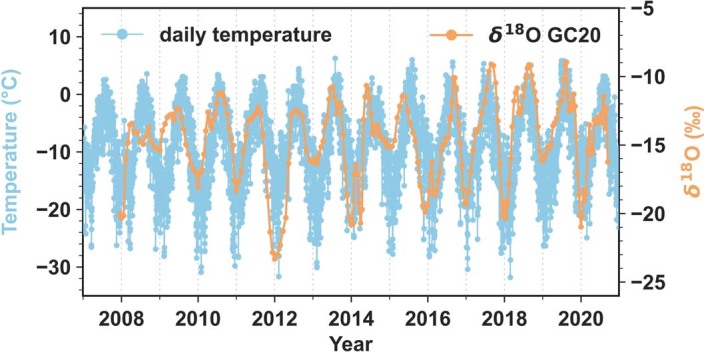
Comparison of δ^18^O and temperatures at the drill site. The Grand Combin firn core 2020 (GC20) δ^18^O record generally follows the temperature variations during the period 2008–2020.

**Extended Data Fig. 2:**
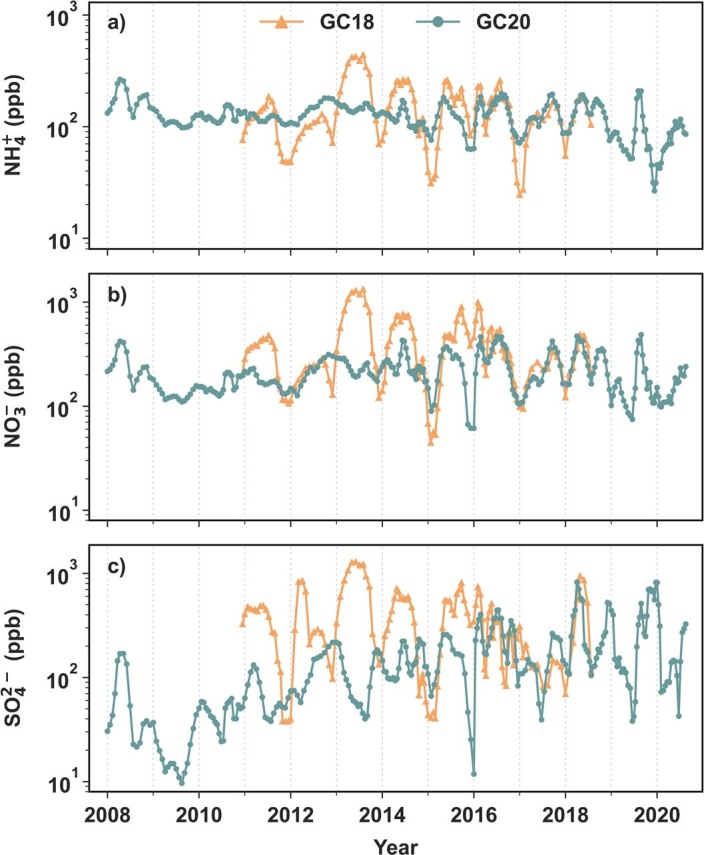
Major ion concentrations of the Grand Combin 2018 (GC18) and 2020 (GC20) firn cores displayed on a logarithmic scale. Comparisons of the records from the two sites on a logarithmic scale is shown to highlight the long-term trends of the concentrations of **a**) NH_4_^+^, **b**) NO_3_^−^, and **c**) SO_4_^2−^.

**Extended Data Fig. 3:**
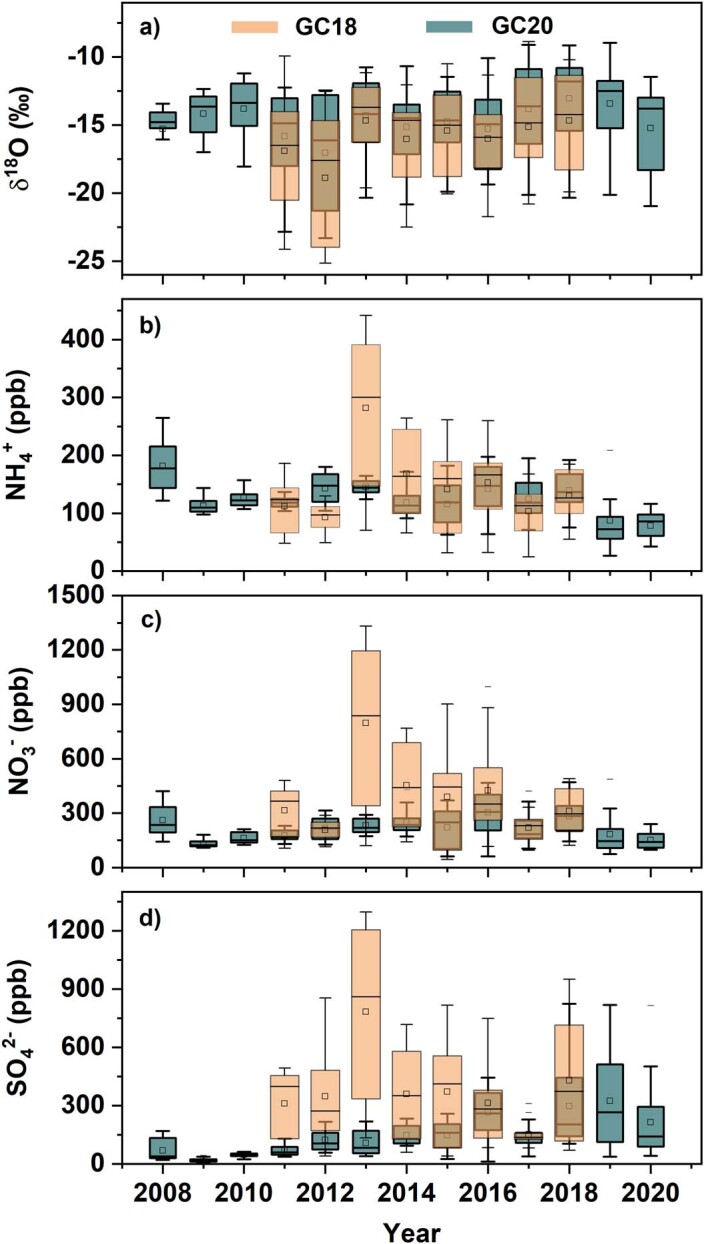
Boxplots of annual Grand Combin 2018 (GC18) and 2020 (GC20) δ^18^O and major ion concentrations. Comparison of the annual box plots showing the minimum, first quartile, mean (square), median, third quartile, and maximum of **a**) δ^18^O and the concentrations of **b**) NH_4_^+^, **c**) NO_3_^−^, and **d**) SO_4_^2−^. Number of samples per year: GC18: n = 16, 12, 12, 20, 18, 23, 10, 9 (2011–2018); GC20: n = 15, 16, 21, 16, 13, 19, 27, 15, 27, 17, 25, 27, 19 (2008–2020).

**Extended Data Fig. 4:**
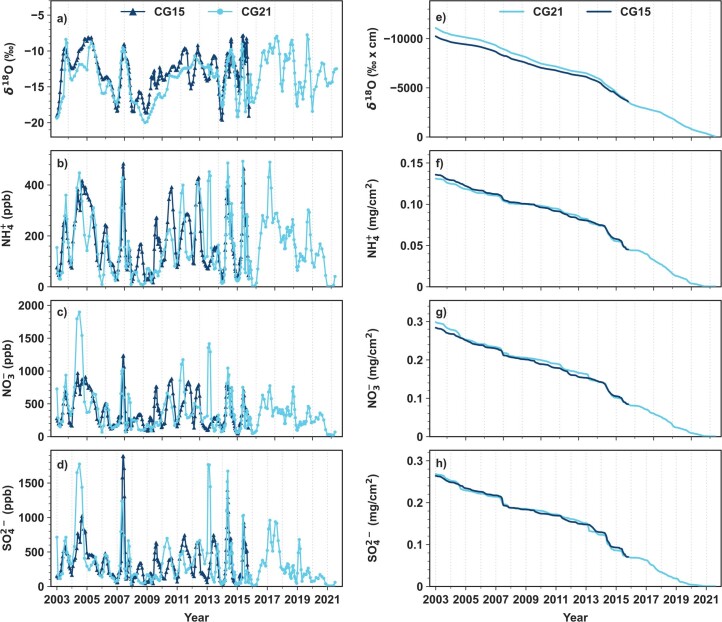
Comparison of δ^18^O and major ion concentrations between two ice cores from Colle Gnifetti to demonstrate their spatial variability. Records from two Colle Gnifetti ice cores collected in 2015 (CG15^16^, period 2003−2015) and 2021 (CG21, period 2003–2021, unpublished) within ca. 20 m distance (4450 m a.s.l., Monte Rosa Massif) are presented: **a**) δ^18^O, concentrations of **b**) NH_4_^+^, **c**) NO_3_^-^ and **d**) SO_4_^2−^ and **e**) cumulative δ^18^O signal and cumulative deposition fluxes for **f**) NH_4_^+^, **g**) NO_3_^−^ and, **h**) SO_4_^2−^. The Colle Gnifetti is a cold glacier site, not influenced by meltwater disturbance, located ca. 45 km east from the Corbassière glacier. This site is prone to wind erosion mainly removing dry winter snow and thus, a high spatial variability is expected. Accordingly, the major ion concentration records of the two Colle Gnifetti cores in the overlapping period 2003-2015 do partially show interannual differences, but the long-term trends and the cumulative deposition fluxes perfectly agree. The δ^18^O signal as a temperature proxy is more sensitive to the amount of winter snow removal than the major ions, which are transported as aerosol particles by convection in summer. This is reflected in the slightly diverging cumulative δ^18^O records with depth (time). Nevertheless, the example of the Colle Gnifetti site illustrates that even if there is some spatial variability of major ion concentrations, cumulative deposition fluxes between close-by sites commonly agree.

**Extended Data Fig. 5:**
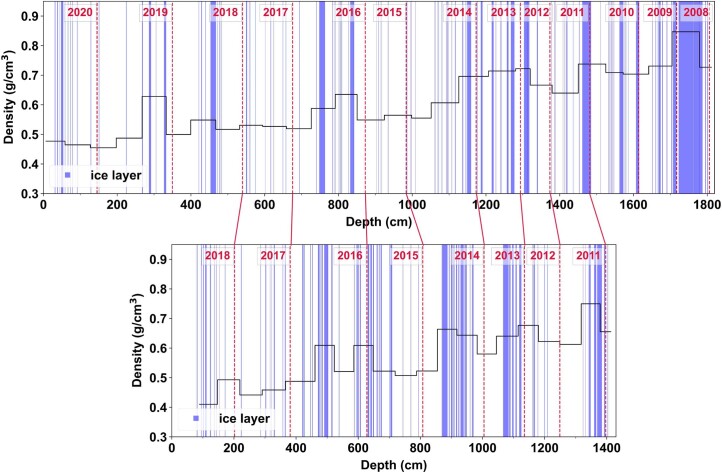
Density profiles and ice layers illustrated for the two Grand Combin cores. Density profiles (black lines) of the 2020 (GC20, upper panel) and 2018 (GC18, lower panel) cores are shown along with the observed refrozen infiltration ice layers (blue), and attributed years.

In this Brief Communication we compare the major ion concentration and stable oxygen isotope ratio in water (δ^18^O) records from two firn cores drilled on Corbassière glacier in 2018 (14.1-m length, GC18) and 2020 (18.1 m, GC20), respectively. Firn is the intermediate stage in the transformation of snow to glacier ice and is porous, whereas glacier ice is nearly impermeable to liquid water. The δ^18^O signals of both firn cores are in excellent agreement for the overlapping period 2011–2018 (Fig. 2a) and generally follow the seasonal temperature trend (Extended Data Fig. 1). In the common, upper part (2016–2018), similar major ion concentrations are observed for ammonium (NH_4_^+^), nitrate (NO_3_^−^) and sulfate (SO_4_^2−^). In the part of GC20 below 8-m depth (year 2016), ion concentrations are generally substantially smoother, and the annual mean values are lower (Fig. 2b–d; Extended Data Fig. 2, same data on a log scale; Extended Data Fig. 3). The annual means began to be significantly lower at the 95% confidence level in 2016 (NO_3_^−^), 2015 (SO_4_^2−^) and 2014 (NH_4_^+^), respectively (except for NH_4_^+^ in 2011, and NO_3_^−^ in 2011 and 2012; Extended Data Table 1). These differences are even more pronounced in the cumulative deposition fluxes, diverging from the year 2016 (NO_3_^−^ and SO_4_^2−^) and 2014 (NH_4_^+^) back in time (Fig. 2f–h). The observed discrepancy cannot be explained by the spatial variability of the ion profiles, which is typically much smaller at high-altitude glaciers. This is illustrated in the example of the Colle Gnifetti glacier, because no such data exist for the summit plateau of the Corbassière glacier (Extended Data Fig. 4). Instead, we attribute the observed deviation to melt-induced elution processes. The lower cumulative deposition fluxes in the GC20 core suggest that ions were either relocated with meltwater to even deeper layers or removed entirely from the archive with the percolating meltwater (runoff). In contrast, δ^18^O as the matrix component was not yet affected by the amount of melt (Fig. 2e). Among the ions, SO_4_^2−^ shows the strongest depletion (Fig. 2h), as expected based on previous observations^19–21^. Those concluded that NH_4_^+^, and to some degree also NO_3_^−^, are preferentially incorporated inside the ice crystal structure, whereas SO_4_^2−^ is enriched at the firn grain surfaces^20,21^, thus being especially sensitive to relocation (or removal) by percolating meltwater as observed here. Further evidence for melt processes is the presence of refrozen infiltration ice layers from the top in both cores (Extended Data Fig. 5), producing positive outliers in the density profiles. Overall, the ice layer records of both cores show similar features, although thicker ice layers appear already in the top part of GC20, in contrast to the GC18 core. Wet firn conditions were encountered below 14-m depth during drilling in 2020, most probably related to the presence of a nearly 1-m-thick ice layer at 17-m depth, preventing further downward percolation of meltwater. We assume that the meltwater ran off along this impermeable layer, because ions were not enriched at this depth.

Daily air temperatures increased continuously over the period from 2007 to 2020, and the trend is significant (Mann–Kendall test: slope = 0.000,27, *P* < 0.000,07; Fig. 1c). In addition, after 2015 there is a shift to a significantly higher sum of air temperatures above the melting point per day (positive degree-day sum (pdd), Fig. 1d; average 2015–2020, 0.24; significance of shift, *t*-test, *P* < 0.000002) and a lower fraction of the day with negative temperatures (ndd, Fig. 1e; average 2015–2020, 0.89; *t*-test, *P* < 0.00000005) compared to the years before (2007–2014; pdd average, 0.15; ndd average, 0.93). This regime shift implies that there was more energy available for melting and fewer days with temperatures below 0 °C in the years 2015 to 2020 than before.

We hypothesize that alterations in the preservation of chemical impurity signals observed between 2018 and 2020 in the Grand Combin firn cores were caused by a combination of the following factors:

- The active layer describes the uppermost firn layer, in which thermal heat transfer from the seasonally varying surface air temperatures causes corresponding seasonal temperature changes (decreasing in amplitude with depth)^22^. The active layer depth at the Grand Combin drilling site is unknown. On Colle Gnifetti (312 m higher, 45 km east of Grand Combin), borehole temperatures suggest an active layer thickness of ~10–20 m (ref. ^23^). On Col du Dôme (112 m higher, 35 km southwest of Grand Combin), borehole temperatures below a depth of 20 m are consistent^24^. We therefore assume an active layer depth at the Grand Combin drilling site of ~10–20 m. Meltwater formed at the surface in summer percolated through this part without causing a detectable alteration in the signal, because cold from the previous winter was stored in this layer. The quantity of meltwater formed at the surface and the corresponding available thermal energy was not sufficient to raise temperatures to 0 °C in this layer, therefore not causing additional melt in the firn and no detectable alteration of the recorded proxy signals (Fig. 2b–d, back to 2016 at 8-m depth in GC20).
- In the porous firn of the active layer, preferential percolation in channels might have occurred, thus leaving a vast area of the firn (including the area of the GC20 ice core) unaffected. Below, preferential percolation is less likely because of increasing density and lower porosity.
- Below the active layer, summer meltwater refroze, as indicated by the thick ice layer, thereby releasing latent heat. At this depth, where the firn is isolated from the cold winter conditions by the active layer above, this additional influx of heat led to a temperature well above the average air temperature, leading to meltwater accumulation^25^. A high water content at this depth was confirmed by the observation of very wet firn during drilling. The high water content enabled percolation of water to even deeper parts, or, considering porosity, eventual horizontal runoff along the firn–ice transition. The water-soluble chemical impurities were thereby continuously removed.
- With the recent air temperature increase, surface meltwater has formed every summer. Firn layers older than one year have therefore experienced more than one percolation event, leading to a cumulative effect (a basic principle of an increasing number of chromatography elution cycles with time). This process explains why, even though the temperature shift had already occurred in 2015, no effect was apparent in the 2018 core.

Our findings underline the delicate condition even glaciers at the highest altitudes in the Alps have reached due to climate warming. Within a short period of only two years, the summit plateau of the Corbassière glacier has reached a tipping point, leaving this natural archive unsuitable for reconstructing major aerosol components at least for the recent period covered by the firn part.

## Methods

Altogether, four firn cores were collected on the summit plateau of the Corbassière Glacier, in the Grand Combin massif, one on 5 October 2018 (GC18; 7.29269° E, 45.93814° N; 4,138 m a.s.l., to a depth of 14.1 m) and three from 15 to 19 September 2020 (7.29265° E, 45.9378° N; 4,123 m a.s.l., to depths of 17.6 m, 18.1 m and 25.3 m) as part of the ICE MEMORY initiative^15^. The firn core GC18 and blank ice were cut in Venice at the Institute of Polar Sciences. The 18.1-m firn core (GC20) and blank ice were cut in the cold room (−20 °C) at the Paul Scherrer Institut (PSI). All the samples were analysed at PSI.

Concentrations of the major ions (for example, NH_4_^+^, NO_3_^−^ and SO_4_^2−^, discussed in this study) were determined with ion chromatography (IC, 850 Professional, Metrohm)^20^. Aliquots of the samples were used to analyse the stable isotopes in water (δD and δ^18^O) by cavity ring-down spectroscopy (WS-CR DS, L2130-*I* Analyser, Picarro)^20^. The firn cores were dated by annual layer counting, considering seasonal variations in δ^18^O and NH_4_^+^ concentration.

To estimate the temperatures at the Grand Combin drill site, the instrumental temperature record from Capanna Margherita^27^ (Monte Rosa Massif, 4,560 m.a.s.l., 45 km from Grand Combin) was used, because the altitude difference is the smallest of all surrounding stations. Temperatures were corrected with a lapse rate of 0.599 °C per 100 m for a vertical elevation difference of 436 m. The lapse rate was obtained by linear extrapolation of the instrumental temperatures from nearby high-altitude stations in the Swiss Alps (Piz Corvatsch, Gornergrat, Col du Grand St-Bernhard, Jungfraujoch; IDAWEB, MeteoSwiss) over the period 2007–2020 within a radius of 200 km, including those from Capanna Margherita. The obtained rate agrees well with the environmental lapse rate (0.65 °C per 100 m), which has been shown to vary regionally^28^. Any gaps in the derived Grand Combin temperature series were filled using quantile mapping^29,30^ of the hourly data from seven stations (additionally Stockhorn, Mont Blanc Colle Major and Mont Blanc Observatoire Vallot). Missing values in the initial series were replaced by the corresponding arithmetic means of the composite series.

Using the derived Grand Combin temperature record, we calculated the positive degree-day sum (pdd)^31^ and the fraction of the day with negative temperature (ndd)^31^. Using pdd and ndd is a simple approach for a melt model relying solely on temperature, as there are no radiation data available for the drill site.

## Online content

Any methods, additional references, Nature Portfolio reporting summaries, source data, extended data, supplementary information, acknowledgements, peer review information; details of author contributions and competing interests; and statements of data and code availability are available at 10.1038/s41561-023-01366-1.

## Figures and Tables

**Extended Data Table 1:**
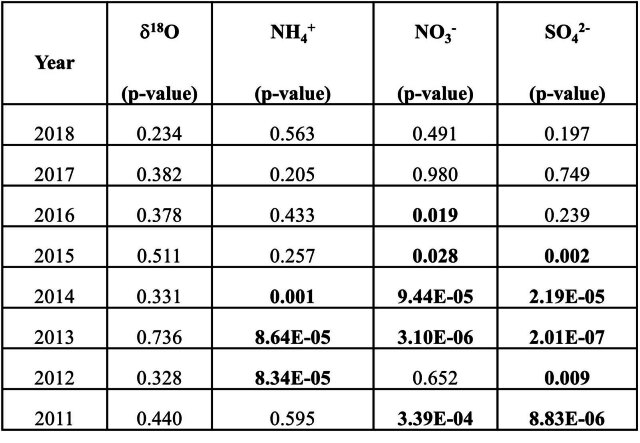
Results of the two-sided t-test, performed to analyse differences between the Grand Combin 2018 (GC18) and 2020 (GC20) firn core annual data

p-values indicate, if the annual means of δ^18^O and the concentrations of NH_4_^+^, NO_3_^-^ and SO_4_^2−^ are significantly different at the 95% confidence level (p-value < 0.05, bold). For the number of samples per year (n-values), see figure caption Extended Data Fig. 3.

## Data Availability

The data presented in this work are available at NOAA World Data Service for Paleoclimatology (WDS-Paleo) at https://www.ncei.noaa.gov/access/paleo-search/study/38739.
